# DEPP Deficiency Contributes to Browning of White Adipose Tissue

**DOI:** 10.3390/ijms23126563

**Published:** 2022-06-12

**Authors:** Fusheng Guo, Yanlin Zhu, Yaping Han, Xuhui Feng, Zhifu Pan, Ying He, Yong Li, Lihua Jin

**Affiliations:** 1 State Key Laboratory of Cellular Stress Biology, School of Life Sciences, Xiamen University, Xiamen 361102, China; guofusheng@pku.edu.cn (F.G.); zhuyanlin416@163.com (Y.Z.); yapinghan@mail.tsinghua.edu.cn (Y.H.); fengxuhui1010@163.com (X.F.); panzhifu@kexing.com (Z.P.); 2 Laboratory Animal Center, Xiamen University, Xiamen 361102, China; hey@xmu.edu.cn; 3 Arthur Riggs Diabetes & Metabolism Research Institute, Beckman Research Institute, City of Hope National Medical Center, Duarte, CA 91010, USA

**Keywords:** decidual protein induced by progesterone, white adipose tissue, brown adipose tissue, thermogenesis, insulin sensitivity, obesity, diabetes

## Abstract

Decidual protein induced by progesterone (DEPP) was originally identified as a modulator in the process of decidualization in the endometrium. Here, we define that DEPP is involved in adipose tissue thermogenesis, which contributes to metabolic regulation. Knockdown of DEPP suppressed adipocyte differentiation and lipid accumulation in 3T3-L1 cells, induced expression of brown adipose tissue (BAT) markers in primary brown adipocyte and induced mouse embryonic fibroblasts (MEFs) differentiation to brown adipocytes. Moreover, DEPP deficiency in mice induced white adipocyte browning and enhanced BAT activity. Cold exposure stimulated more browning of white adipose tissue (WAT) and maintained higher body temperature in DEPP knockout mice compared to that in wild-type control mice. DEPP deficiency also protected mice against high-fat-diet-induced insulin resistance. Mechanistic studies demonstrated that DEPP competitively binds SIRT1, inhibiting the interaction between peroxisome proliferator-activated receptor gamma (PPARγ) and Sirtuin 1 (SIRT1). Collectively, these findings suggest that DEPP plays a crucial role in orchestrating thermogenesis through regulating adipocyte programs and thus might be a potential target for the treatment of metabolic disorders.

## 1. Introduction

Adipose tissues are important metabolic organs, playing irreplaceable roles in regulating metabolic homeostasis, such as thermogenesis and insulin sensitivity. White adipose tissue (WAT) stores energy as lipids and exhibits heat insulation function. On the contrary, brown adipose tissue (BAT) dissipates energy as heat with nonshivering thermogenesis. Compared to white adipocytes, brown adipocytes are more efficient in uncoupling mitochondrial electron transport from ATP synthesis by permeabilizing the inner mitochondrial membrane to allow the intermembrane proton leakage back to the mitochondrial matrix, mainly through uncoupling protein-1 (UCP1) [1]. There is a third kind of adipocyte, brown adipocyte-like cells (known as “beige” or “brite” adipocytes) that can be induced within WAT under a certain stimulus. Beige adipocytes are morphologically indistinguishable from brown adipocytes and function similarly in thermogenesis. During aging and the development of obesity, adiposity increases, whereas BAT depots and activity that can be indicated by UCP1 expression decline [2]. On the contrary, interventions including cold exposure, caloric restriction, physical activity and bariatric surgery are all accompanied by WAT browning [3]. Therefore, promoting the conversion of WAT to beige fat (or named white adipocyte browning) is a promising therapeutic strategy for controlling metabolic diseases [3].

Evidence has demonstrated that browning of WAT can be induced by some hormones and cytokines, such as irisin [4] and fibroblast growth factor 21 (Fgf21) [5], as well as by transcriptional modulation through PR domain containing 16 (Prdm16) [6], transcriptional mediators/intermediary factor 2 (TIF2) [7], Forkhead Box C2 (FoxC2) [8], receptor-interacting protein 140 (RIP140) [9], eIF4E-binding protein 1 (4E-BP1) [10], pRb and p107 [11]. Notably, in addition to improving insulin sensitivity [12,13], activation of the transcriptional factor PPARγ can also induce browning of white adipocytes by inhibiting the WAT-selective gene cascade and promoting BAT-selective gene cluster expression [12]. SIRT1 is a histone deacetylase that also functions as a transcription factor for many different physiological processes. Activation of SIRT1 increases mitochondrial biogenesis and thermogenesis, indicating the critical role of SIRT1 in adipocyte browning [14,15]. SIRT1 gain-of-function mimics the PPARγ activation in alleviating insulin resistance [16]. Importantly, SIRT1 has been shown to directly interact with PPARγ, and SIRT1-mediated PPARγ deacetylation effectively promotes WAT browning [17]. These results suggest an important role of the SIRT1–PPARγ interaction in regulating energy homeostasis.

Decidual protein induced by progesterone (DEPP) was first isolated from a progesterone-induced endometrial stromal cell cDNA library [18]. Earlier reports suggest that DEPP modulates the effects of progesterone during decidualization [18]. DEPP is expressed in endothelial cells of peripheral tissues, but not in atrial or ventricular endocardial cells of the heart, providing a marker gene to discriminate between these two cell types. DEPP is also found to be upregulated in subsets of endothelial cells in settings of adult neovascularization, including tumor angiogenesis [19]. Although DEPP has been found expressing in various human tissues, its physiological function remains unclear. Notably, DEPP expression was later found highly induced in WAT by fasting in mice [20]. Moreover, DEPP expression is increased in 3T3-L1 cells in a differentiation-dependent manner similarly to WAT-selective genes, such as adiponectin and aquaporin 7 [20]. These results suggest a role of DEPP in adipocytes; however, the detailed function of DEPP in adipocytes and the underlying molecular mechanism remain unclear.

In this study, we generated a DEPP-knockout (KO) mouse model (Depp^−/−^) and found that DEPP deficiency improved the browning of white adipocytes. Competitive binding of SIRT1 with PPARγ may be one of the underlying mechanisms.

## 2. Results

### 2.1. DEPP Deficiency Reduces Lipid Storage in White Adipocytes and Induces Browning of Adipocytes In Vitro

After inducing the differentiation of preadipocyte 3T3-L1 cells, we measured the relative expression of Depp and some genes related to white adipocyte differentiation and adipogenesis, including Pparg, CCAAT enhancer-binding protein alpha (Cebpa) and beta (Cebpb) using qPCR. The result showed that Depp highly expressed in the differentiated 3T3-L1 cells like the other tested genes (Figure S1a, see Supplementary Materials). We also measured the mRNA and protein levels of DEPP at different stages during the differentiation of 3T3-L1 cells and observed that along with the differentiation that was indicated by the expression level of adiponectin, both the mRNA and protein levels of DEPP showed an increasing manner (Supplemental Figure S1b–e), which is consistent with a previous report [20].

**Figure 1:**
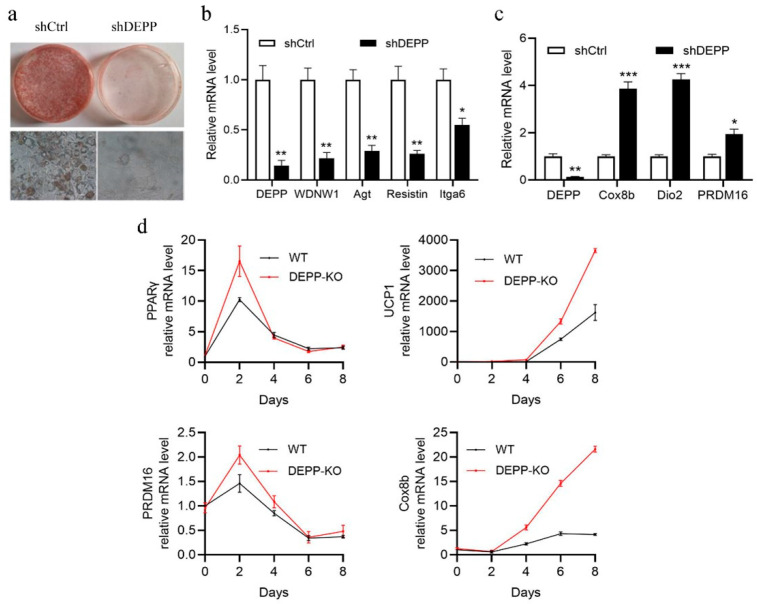
DEPP deficiency reduced lipid storage in white adipocytes and induced browning of adipocytes. (**a**,**b**) 3T3-L1 cells with Depp knockdown by shRNA (shDepp) and their control cells with schrambled shRNA (shCtrl) were induced differentiation with the hormone cocktail dexamethasone–methylisobutylxanthine–insulin (DMI). (**a**) Oil Red O staining images and their magnified images are shown. (**b**) Relative mRNA levels of white adipocyte marker genes. (**c**) Primary brown adipocytes were isolated from the brown adipose tissue in the scapular region of newborn mice. DEPP was silenced by Depp shRNA. Relative mRNA levels of brown adipocyte marker genes were determined after cell differentiation. (**d**) MEFs isolated from DEPP knockout (KO) mice and wild-type (WT) littermates were induced for differentiation. The dynamic mRNA level of brown adipocyte marker genes was measured at 0, 2, 4, 6 and 8 days of differentiation. The data of b, c and d were obtained from triplicate biological samples. The results are presented as the means ± SD, two-tailed Student’s *t*-test: * *p* < 0.05, ** *p* < 0.01, *** *p* < 0.001.

To figure out the role of DEPP in adipocyte differentiation, we stably silenced Depp expression in 3T3-L1 cells mediated by lentivirus with shRNA-Depp and used scrambled shRNA as a control. Among the designed Depp shRNAs, shDepp-6 showed the most effectiveness in Depp knockdown (KD) in 3T3-L1 cells (Figure S2); therefore, we selected this shRNA for the following experiments. After inducing differentiation, compared to that in control (shCtrl) 3T3-L1 cells, Depp KD cells showed significantly delayed adipocyte differentiation with dramatically reduced lipid accumulation (Figure 1a). Accordingly, lower expression of white adipocyte markers, including Wdnm1, Agt, Resistin and Itga6, were observed (Figure 1b). We then checked the effect of Depp KD in brown adipocytes. The primary brown adipocytes were isolated from newborn mice and infected with the shDepp lentivirus, then induced to differentiation. Interestingly, along with the KD of Depp, the expressions of brown adipocyte marker genes such as Dio2, Prdm16 and Cox8b were significantly induced (Figure 1c). These results suggest a potential role of DEPP in white adipocyte browning.

Next, we created a Depp KO mouse model by the transcription-activator-like effector nucleases (TALEN) technique to investigate the physiological function of DEPP. The loss of five bases on the second exon resulted in a frameshift mutation to achieve the purpose of DEPP KO (Figure S3). After getting the homologous DEPP KO (Depp^−/−^) mice, we isolated the mouse embryonic fibroblasts (MEFs) and induced differentiation to adipocytes. Consistently with what we observed in 3T3-L1 cells and primary brown adipocytes, MEFs from Depp^−/−^ mice showed significantly induced expression of brown adipocyte marker genes, including Ucp1, Prdm16, PPARγ and cytochrome c oxidase subunit 8B (Cox8b) (Figure 1d), indicating that DEPP negatively regulated the brown adipocyte marker genes, further suggesting that DEPP deficiency may induce adipocyte browning.

### 2.2. DEPP Deficiency Enhances BAT Activity and Induces White Adipocyte Browning in Mice

**Figure 2:**
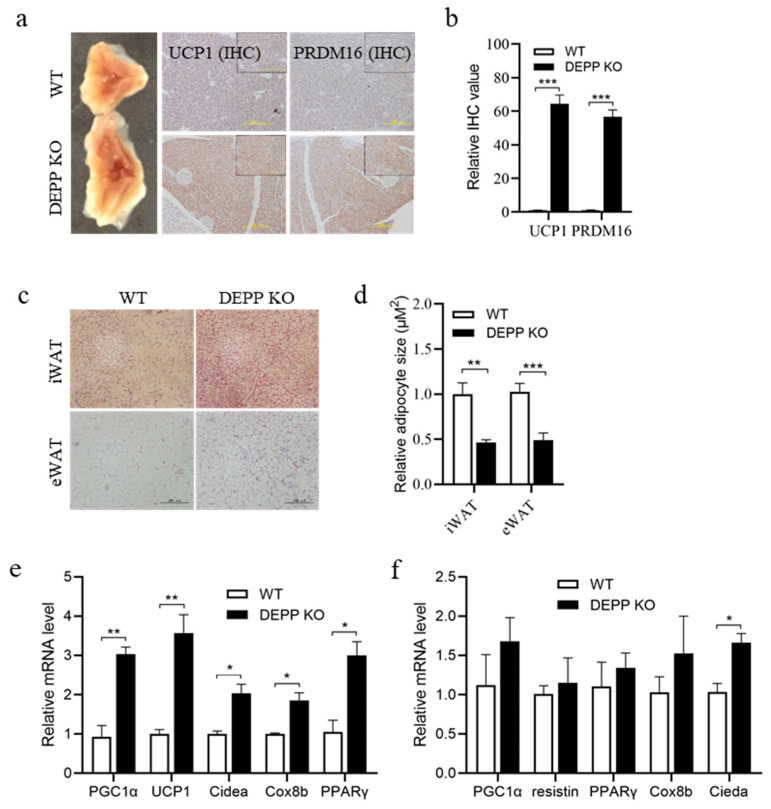
DEPP deficiency enhanced BAT activity and induced white adipocyte browning in mice. (**a**) Representative images of the scapular region of BAT from newborn DEPP KO and wild-type (WT) littermate mice, and representative images of immunohistochemical (IHC) stained BAT sections against UCP1 and PRDEM16, respectively. Scale bar, 200 μM. (**b**) Relative quantitative value of UCP1 and PRDM16 expression in (**a**). (**c**) Representative images of H&E-stained iWAT and eWAT sections from DEPP KO and WT littermate mice. Scale bar, 100 μM. (**d**) Relative adipocyte size of iWAT and eWAT calculated from (**c**). (**e**,**f**) Relative mRNA levels of BAT marker genes in iWAT (**e**) and eWAT (**f**) from DEPP KO and WT littermates. *n* = 6 biological replicates; the results are presented as the means ± SD. Two-tailed Student’s *t*-test: * *p* < 0.05, ** *p* < 0.01, *** *p* < 0.001.

As expected, we observed larger BAT mass in newborn Depp^−/−^ mice than that in wild-type (WT) littermates (Figure 2a). Immunohistochemical examination using antibodies against UCP1 and PRDM16 showed higher expression levels of these brown adipocyte markers (Figure 2a,b). Depp^−/−^ mice at 6–8-weeks showed smaller adipocyte size in inguinal WAT (iWAT) and epididymal WAT (eWAT) sections stained by hematoxylin and eosin (HE) (Figure 2c,d). Importantly, significantly higher expressions of BAT marker genes were observed in iWAT from Depp^−/−^ mice than in that from WT littermates (Figure 2e). Similar trends were also observed in eWAT (Figure 2f). These results suggest that DEPP deficiency enhanced BAT activity and induced WAT browning.

### 2.3. Cold Exposure Stimulates More Browning of WAT in DEPP KO Mice

**Figure 3:**
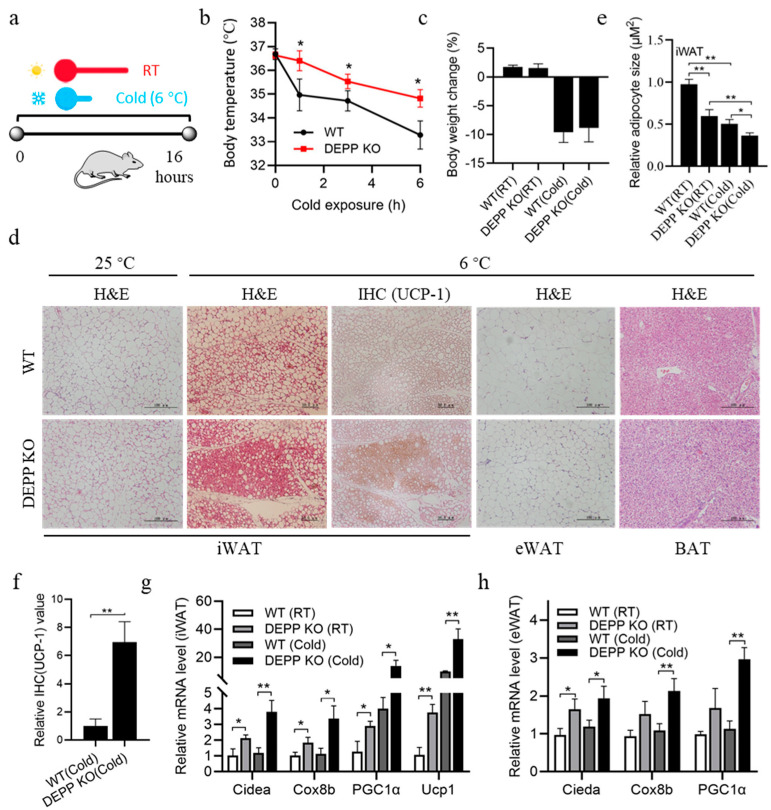
Cold exposure stimulated more browning of WAT in DEPP KO mice than in WT litermates. (**a**) Experimental flow chart for overnight cold exposure treatment. (**b**) Dynamic body temperature changes of DEPP KO and WT littermates during cold exposure. (**c**) Body weight changes at 16 h of cold exposure. (**d**) Representative images of H&E-stained iWAT, eWAT and BAT sections of DEPP KO and WT littermates at room temperature (RT) or after 16 h of cold exposure, IHC stained iWAT sections against UCP1 are also shown. Scale bar indicates 50 or 100 μM. (**e**) Relative adipocyte size calculated from iWAT sections in (**d**). (**f**) Relative quantitative values of UCP1 expression in IHC-stained iWAT in (**d**). (**g**) Relative mRNA levels of BAT marker genes of iAWT. (**h**) Relative mRNA levels of BAT marker genes in eWAT. *n* = 6 biological replicates; the results are presented as the means ± SD, by two-tailed Student’s *t*-test (**b**,**f**) and one-way ANOVA with Tukey’s post hoc test (**c**,**e**,**g**,**h**): * *p* < 0.05, ** *p* < 0.01.

Exposure to a cold environment will stimulate thermogenesis of BAT and promote the browning of white adipocytes. To further confirm the role of DEPP in thermogenesis, we put mice under an acute overnight cold treatment (6 °C for 16 h) (Figure 3a). As expected, cold exposure decreased the body temperature of WT and Depp^−/−^ mice; however, Depp^−/−^ mice maintained a significantly higher body temperature than WT mice did (Figure 3b) without a significant difference in body weight change (Figure 3c). We then examined the adipose tissues of these mice. Cold exposure decreased the size of white adipocytes indicated by the H&E-stained iWAT sections in WT and Depp^−/−^ mice, while reduced size was observed in more adipocytes in Depp^−/−^ mice (Figure 3d,e). Moreover, immunohistochemistry (IHC) staining showed more UCP1 in the iWAT sections of Depp^−/−^ mice (Figure 3d,f), suggesting more browning of WAT in Depp^−/−^ mice under a cold stimulus. Smaller adipocyte size was also observed in eWAT sections from Depp^−/−^ mice than that in WT mice, while there was no difference in BAT sections (Figure 3d). Consistent with the phenotype above, induced expressions of BAT marker genes were observed in the iWAT of WT and Depp^−/−^ mice after cold stimulus, while those in Depp^−/−^ mice showed a significantly higher induction than in that WT mice (Figure 3g). Similar changes with a lower extent were observed in the expressions of these genes in the eWAT of these mice (Figure 3h). These results suggest that DEPP deficiency effectively induced white adipocyte browning and thermogenesis under a cold stimulus in vivo.

### 2.4. DEPP Deficiency Improves Insulin Sensitivity under HFD Feeding in Mice

**Figure 4:**
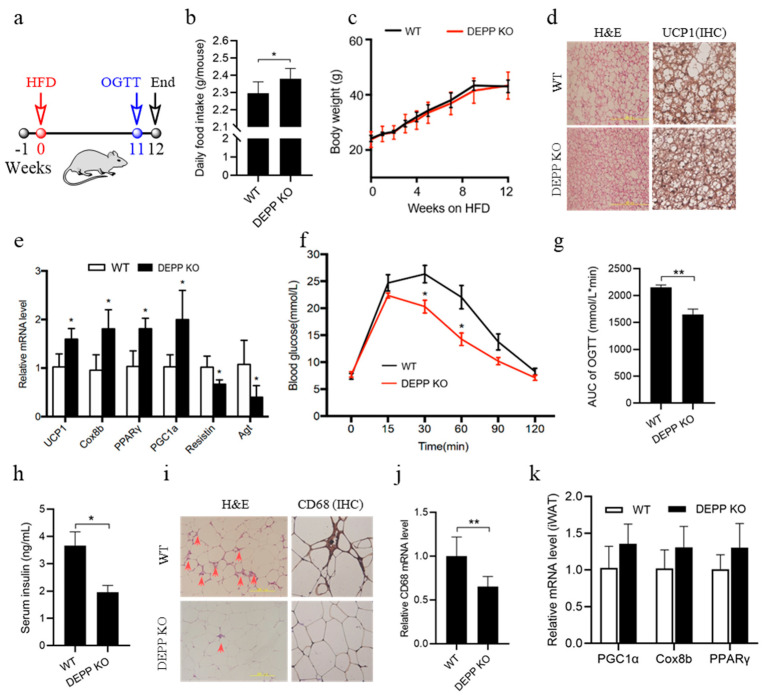
DEPP deficiency improved insulin sensitivity under HFD feeding in mice. (**a**) A high-fat diet (HFD) experimental flow chart for 12 weeks. (**b**) Food intake. (**c**) Body weight. For (**d**,**e**,**h**–**k**), samples were collected at the end timepoint of HFD feeding. (**d**) Representative images of H&E-stained BAT sections and IHC-stained BAT sections against UCP1. Scale bar, 200 μM. (**e**) Relative mRNA level of genes in BAT. (**f**) Oral glucose tolerance test (OGTT) at the 11-week timepoint during HFD feeding. (**g**) Quantitative area under the curve (AUC) of OGTT in (**f**). (**h**) Fasting serum insulin level. (**i**) Representative images of H&E-stained eWAT sections and IHC-stained eWAT sections against CD68. Scale bar, 200 μM. (**j**) Relative mRNA level of CD68 in eWAT. (**k**) Relative mRNA level in iWAT. The results are presented as the means ± SD. Two-tailed Student’s *t*-test: * *p* < 0.05, ** *p* < 0.01.

Excessive fat intake is the main reason inducing insulin resistance and adipose inflammation. We examined the effect of DEPP deficiency in mice under HFD feeding. Depp^−/−^ mice and WT littermates were fed HFD from 6–8-weeks-old for 12 weeks (Figure 4a). Interestingly Depp^−/−^ mice exhibited more food intake than WT littermates did (Figure 4b), while keeping a similar body weight (Figure 4c). This may be due to the more activated BAT in Depp^−/−^ mice indicated by smaller adipocyte size and more UCP1 expression (Figure 4d). DEPP knockout increased the expression of thermogenesis genes in BAT and decreased the expression of genes related to brown adipocyte whitening (Figure 4e), leading to increased BAT activity and alleviated whitening of brown adipocytes caused by the excessive fat intake. Accordingly, Depp^−/−^ mice showed improved glucose tolerance (Figure 4f,g) and insulin sensitivity (Figure 4h).

Dysfunction of BAT and browning of white adipocytes leads to chronic inflammation in obesity, and inflammation due to infiltration by macrophages and other immune cells contributes greatly to WAT pathophysiology in adiposity including insulin resistance [21]. As shown in the results, there were much fewer inflammatory macrophages in the WAT of Depp^−/−^ mice that could be confirmed by IHC staining against CD68 (Figure 4i) and CD68 mRNA identification (Figure 4j), a routinely used histochemical/cytochemical marker of inflammation associated with the involvement of monocytes/macrophages. There was only a trend but not significant changes in the expression of browning markers in iWAT (Figure 4k). These data suggested that DEPP deficiency improved insulin sensitivity by activating thermogenesis and energy expenditure in BAT.

### 2.5. DEPP Competitively Binds SIRT1 with PPARγ

**Figure 5:**
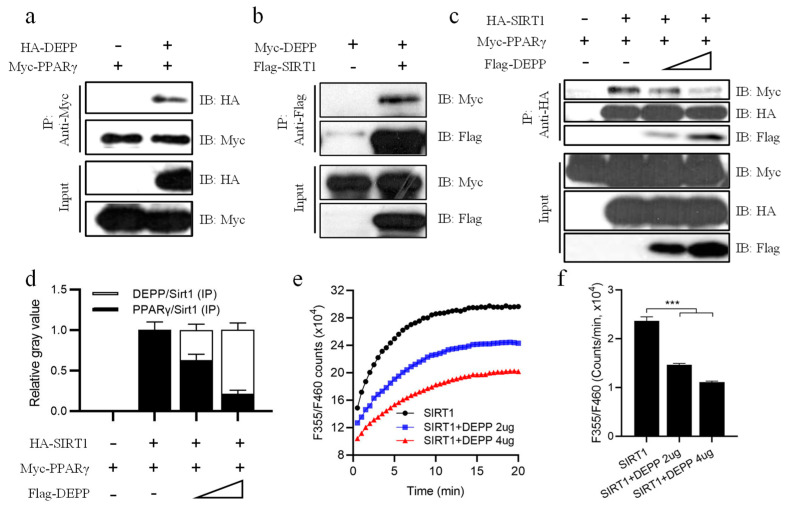
DEPP intercepted the interaction between SIRT1 and PPARγ. (**a**) pCMV-HA-DEPP and pCMV-Myc-PPARγ plasmids were co-transfected into HEK293T; then, cell lysates were immunoprecipitated with an anti-Myc antibody and immunoblotted with indicated antibodies. (**b**) pCMV-Myc-DEPP and pCMV-Flag-SIRT1 plasmids were co-transfected into HEK293T; then, cell lysates were immunoprecipitated with M2 beads (anti-Flag antibody) and immunoblotted with indicated antibodies. (**c**) pCMV-HA-SIRT1, pCMV-Myc-PPARγ and a gradient number of pCMV-Flag-DEPP plasmids were co-transfected into HEK293T; then, cell lysates were immunoprecipitated with an anti-HA antibody and immunoblotted with indicated antibodies. (**d**) Relative quantitative gray value of Myc-PPARγ (IP)/HA-SIRT1 (IP) and Flag-DEPP (IP)/HA-SIRT1 (IP) in (**c**). (**e**) pCMV-HA-SIRT1 with a gradient amount of plasmid pCMV-HA-DEPP were co-transfected into HEK293T cells, and the cell lysate was used for a deacetylase activity assay determined by a CycLex^®^ SIRT1/Sir2 Deacetylase Fluorometric Assay Kit. (**f**) Reaction rate of SIRT1 enzyme activity in (**e**). *n* = 3 biological replicates; the results are presented as the means ± SD. One-way ANOVA with Tukey’s post hoc test: *** *p* < 0.001.

SIRT1 binds and deacetylates PPARγ to promote the browning of white adipocytes and consequently induces thermogenesis, improves insulin sensitivity and reduces lipid storage in the body [17]. Therefore, enhancing the PPARγ and SIRT1 interaction may be beneficial for inducing thermogenesis and promoting insulin sensitivity. Using a co-immunoprecipitation (co-IP) assay, we found that DEPP could interact not only with PPARγ but also with SIRT1 (Figure 5a,b). Then, we asked if DEPP affected the interaction between PPARγ and SIRT1. A gradient amount of plasmid pCMV-Flag-DEPP was co-transfected together with a constant number of plasmids pCMV-HA-SIRT1 and pCMV-Myc-PPARγ into HEK293T cells. Thirty hours later, the cells were harvested for a co-IP assay. We found that along with the increasing level of DEPP, the PPARγ protein level interacting on SIRT1 gradually decreased (Figure 5c,d), indicating a competitive binding between DEPP and PPARγ on SIRT1.

In order to know whether the interaction of DEPP on SIRT1 affected the activity of SIRT1, we co-transfected pCMV-HA- SIRT1 with a gradient amount of plasmid pCMV-DEPP into cells and measured the deacetylase activity of cell lysate using a CycLex SIRT1/Sir2 Deacetylase Fluorometric Assay Kit. Interestingly, DEPP significantly inhibited SIRT1 deacetylase activity in a dose-dependent manner (Figure 5e,f). These results suggest that by competitively supplanting PPARγ from SIRT1, DEPP destroyed the PPARγ/SIRT1 complex and inhibited the deacetylase activity of SIRT1, which may contribute to the physiological function of DEPP in adipocytes and metabolism.

## 3. Discussion

Overaccumulation of fat in WAT causes obesity and a series of complications, including T2D, NASH, cardiovascular diseases, and other health problems. Inhibiting lipid accumulation in white adipocytes and inducing white-to-brown adipocyte conversion increases energy expenditure and consequently improves metabolism. In this study, we demonstrated that DEPP is required for adipocyte differentiation and lipid accumulation. DEPP deficiency induces adipocyte browning, thus, Depp^−/−^ mice effectively maintain body temperature under a cold stimulus due to more thermogenesis from BAT and beige fat. In addition, increased energy expenditure by DEPP deficiency also improves insulin sensitivity and adipose inflammation. Competitively binding SIRT1 with PPARγ may contribute to these metabolic benefits. Therefore, DEPP plays a critical role in thermogenesis, energy expenditure and metabolism.

**Figure 6:**
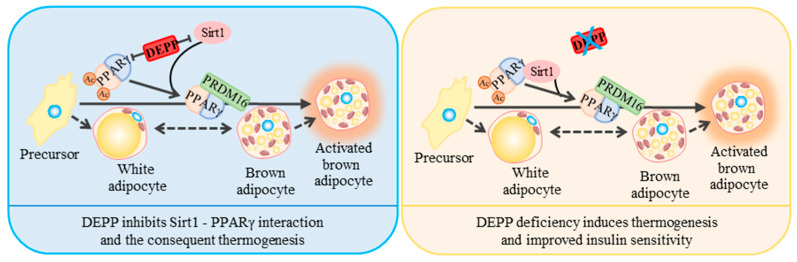
A model of a DEPP-mediated mechanism on regulating adipocyte browning.

Data analysis from a Gene Expression Omnibus (GEO) profile indicated that higher expression of Depp in iWAT is associated with higher weight gain in mice under four weeks’ high-saturated-fat-diet feeding (Figure S4a) [22]. Particularly, higher expression of Depp in WAT is associated with a high triglyceride level in a mice T2D model characterized by obesity, insulin resistance and hyperlipidemia (Figure S4b) [23]. Moreover, there are higher levels of Depp in visceral adipose tissue of obese individuals (Figure S4c) [24]. These data further support the positive correlation between WAT DEPP and metabolic disorders. SIRT1 binds and deacetylates PPARγ, which helps recruit the BAT program coactivator PRDM16 to PPARγ, leading to selective induction of BAT genes associated with browning of white adipocytes and repression of visceral WAT genes associated with insulin resistance [17]. We identified that DEPP could inhibit the PPARγ–SIRT1 interaction, which might prevent the white adipocyte browning progress (Figure 6). The fact that DEPP-deficient MEFs showed more expression of BAT marker genes than WT MEFs did in differentiation supports this hypothesis. Consistently, DEPP KO mice showed enhanced white adipocyte browning compared to that of WT mice. The higher body temperature under a cold stimulus suggested increased thermogenesis by DEPP deficiency. Although measuring the physical activity and indirect calorimetry using a metabolic cage assay may provide more direct data about the energy expenditure of these mice, previous evidence has shown that adenovirus-mediated DEPP overexpression reduces energy expenditure in mice [25], supporting the negative correlation between the level of DEPP and energy expenditure. This group also found that adenovirus-mediated DEPP overexpression reduces food intake in mice [25], which is consistent with our findings that DEPP KO mice exhibit more food intake with more energy expenditure, which might have led to the improved metabolism with unchanged body weight in our study. It has been reported that fasting induced while refeeding decreases DEPP expression in WAT, indicating that DEPP is an insulin-regulatory molecule [20]. It should be noted that fasting reduces energy expenditure [26] and decreases body temperature [27]. Consistently, adenovirus-mediated DEPP overexpression reduces energy expenditure [25], while DEPP deficiency induces energy expenditure and increases body temperature (Figure 3b). These results suggest that DEPP is involved in controlling energy expenditure upon different nutrition conditions. DEPP might play a role in metabolic compensation and negative feedback regulation. That is to say, fasting-induced upregulation of DEPP might prevent the body from further loss of energy, while downregulation of DEPP by refeeding (such as overnutrition under HFD feeding) might prevent the body from excess energy (in the form of fat) accumulation. Together, downregulating DEPP might be a promising strategy to treat obesity by increasing energy expenditure.

In this study, the DEPP KO mouse model by TALENs technique was created as whole-body knockout mice. Previous research showed that DEPP in tissues other than adipose tissue may also be involved in metabolism regulation. For example, fasting induces Depp expression in WAT, lung, skeletal muscle, liver and kidney [20]. By analyzing the dataset from a published GEO profile, we also found that Depp in tissues other than adipose tissue is associated with metabolism disorders. For example, a higher level of Depp was found in skeletal muscle cells in T2D individuals compared to that of a healthy non-T2D group (Figure S5a) [28]. As known, bariatric surgery is the most effective approach to treating obesity that can also efficaciously improve T2D and NAFLD [29,30]. The expression of Depp is significantly downregulated in the liver of individuals with obesity or obesity with NAFLD after bariatric surgery (Figure S5b,c) [30]. These results suggest the important role of DEPP of various tissues in metabolism, and they all show a positive correlation between the level of Depp and metabolic disorders. Further investigation on tissue-specific DEPP KO or overexpressing models would provide more knowledge about the role of DEPP in metabolism.

In summary, these results demonstrate the crucial role of DEPP in regulating adipocyte programs and metabolism, providing new avenues for further understanding the transition between WAT and BAT and a novel potential target for the treatment of obesity and insulin resistance.

## 4. Materials and Methods

### 4.1. mDEPP Protein Purification and Primary Antibody Production

Mouse DEPP (aa 150–205, Genbank# NM_145980.2) was cloned into pET24a plasmid (Novagen, Darmstadt, Germany) tagged with 6 His at the N-terminus. BL21(DE3) cells transfected with a pET24a-6xHis-DEPP plasmid were grown in LB broth at 37 °C to an optical density at 600 nm (OD_600_) of ~0.8 and then induced with 0.1 mM isopropyl 1-thio-β-D-galactopyranoside (IPTG) at 18 °C for 18 h. His-tagged DEPP protein was purified with a HiTrapTM Hp column chelated with NiSO_4_, followed by an SP column (GE Healthcare, Pittsburgh, PA, USA) and then concentrated to 5 mg/mL. Purified DEPP protein was mixed with an immune adjuvant and subcutaneously injected into rabbits. Two months later, serum was collected from the rabbits, and the antibody was purified with Protein A beads. pCMV-HA-mDEPP was transiently transfected into HEK293T cells, and the cell lysates were probed by an anti-HA antibody and the purified anti-DEPP antibody, respectively, by Western blotting analysis to evaluate the generated antibody. The purified antibody could probe DEPP efficiently and selectively in Western blotting analysis (Figure S1e) and was thus used in this study to probe DEPP.

### 4.2. SIRT1 Deacetylase Assay

Plasmids pCMV-HA-DEPP and pCMV-Myc-SIRT1 were co-transfected into HEK293T cells. Thirty hours later, cell lysates were harvested to measure the activity of SIRT1 using a CycLex^®^ SIRT1/Sir2 Deacetylase Fluorometric Assay Kit (MBL international corporation, MA, USA) as instructed.

### 4.3. T3-L1 Culture and Differentiation Induction

3T3-L1 cells were seeded into a 60 mm plate. The day when the cell density reached 100% was recorded as day 0. At day 0, the cell culture medium was changed to fresh complete medium (DMEM with 10% FBS). At day 2, the culture medium was changed to a complete medium containing DMI (1 μM dexamethasone, 0.25 mM 3-isobutyl-1-methylxanthine and 3 μg/mL insulin, Sigma) to induce differentiation. At day 4, the culture medium was replaced with a complete medium containing 3 μg/mL insulin. Cells were cultured for another 4 days and replaced with a fresh medium every 2 days. When cell differentiation was completed at day 8, the cells were harvested for Oil red O staining or RNA, protein isolation for qPCR and Western blotting analysis, respectively.

### 4.4. Mice Embryonic Fibroblasts’ (MEFs) Isolation and Differentiation

Pregnant mice (about 13.5 days of gestation) were euthanized for MEFs’ isolation. Uteri and embryos were separated under aseptic conditions. The head, viscera and limbs of fetal mice were removed, and the trunk was cut into pieces of about 1 mm^3^; then, trypsin was added to digest the tissue sample at 37 °C for 20 min. A complete medium was then added to terminate digestion and centrifuged for 5 min at 1000 rpm and then resuspended and cultured in DMEM containing 10% FBS. For adipocyte differentiation, the medium was supplemented with insulin (10 ug/mL), dexamethasone (1 μM), 3-isobutyl-1-methylxanthine (0.5 mM) and rosiglitazone (0.5 μM) for 2 days. From day 3, the cells were incubated with a fresh medium containing rosiglitazone and insulin. The medium was renewed every 2 days until the end of the experiment [31,32].

### 4.5. Oil Red O Staining

Differentiated cells were gently washed three times with PBS and fixed with 4% paraformaldehyde at 37 °C for 30 min. The cells were washed another three times and kept at room temperature for about 30 min. The cells were then stained with Oil red O at 37 °C for 30 min, washed with ultrapure water and then used for microscopic examination and photography.

### 4.6. Lentivirus-Mediated DEPP Knockdown

shRNA sequences listed in Figure S2 were inserted into a pLV-RNAi vector. DEPP shRNA plasmids or a negative control vector (scramble) were co-transfected with VSV-G, pRSV/REV and pGag-pol into HEK293T cells. Seventy-two hours later, the culture medium was collected, centrifuged at 1300 rpm for 5 min to remove cell debris and then filtered using a 0.45 μm filter to harvest the lentivirus. 3T3-L1 cells at about 60% confluence were infected with the lentivirus. Twenty-four hours after the infection, 5 μg/mL puromycin was added to the medium for 5 days to kill the uninfected cells. The cells were subcultured and qPCR was used to determine whether DEPP was silenced successfully.

### 4.7. CO-IP and Western Blot

pCMV-HA-DEPP and pCMV-Myc-PPARγ, pCMV-HA-DEPP and pCMV-Myc-SIRT1, or pCMV-HA-SIRT1/pCMV-Myc-PPARγ/pCMV-Flag-DEPP were co-transfected into HEK293T cells using a PEI reagent. Thirty hours later, cell lysates were harvested with cold NP-40 lysis buffer for co-precipitation and then Western blotting analysis as described previously [33]. Anti-Flag (Santa Cruz Biotech, Dallas, TX, USA), anti-Myc (9E10) (Santa Cruz Biotech) and anti-HA (Santa Cruz Biotech) antibodies, anti-Flag M2 Affinity Gel (Sigma) and Protein A/G plus agarose beads (Santa Cruz Biotech) were used in the Co-IP and Western blot analysis.

### 4.8. DEPP Knockout Mice Production

The TALEN technique was used to create DEPP KO mice (Cyagen company, Guang Zhou, China). Depp is located in Chromosome 6—NC_000072.7 of C57BL/6J. Depp gene contains 2 exons, and the coding region is located in exon 2 (Figure S3a). The target sequences of the Depp TALENs were as follows: left TGCCCCATTTGCCAACG and right TGCCCCATGTGACAGCTC. The sequence of the spacer was ATTCGGGAAATGTCAGAA. After Golden Gate TALEN assembly, the TALEN mRNAs were transcribed in vitro and injected to fertilized oocytes of mouse. Oocytes were then cultured at 37 °C until transfer into pseudopregnant female mice. We successfully got pups with a 5 nucleotides’ deletion mutation that exhibited loss-of-function for DEPP (Figure S3b).

### 4.9. Animal Treatment

All mice were housed at 22–24 °C with a 12 h/12 h light/dark cycle and provided with water and standard rodent chow ad libitum. All animal experiments in this study were conducted in the barrier facility of Laboratory Animal Center, Xiamen University and approved by the Institutional Animal Use and Care Committee.

For the diet-induced obese (DIO) mouse model, 6–8-week-old male DEPP KO and wild-type (WT) littermate mice were fed a high-fat diet (HFD) (60% fat, 24% carbohydrates and 16% protein based on caloric content, Research Diets D12492) for 12 weeks. Food intake and body weight were monitored once daily. The mice were then euthanized and the tissues and serum were collected for analysis.

For acute cold exposure treatment, 6–8-week-old male DEPP KO and WT littermates were exposed at 6 °C for 16 h [17]. Body temperature was monitored at the first 6 h. After euthanasia, the tissues and serum were collected for analysis.

### 4.10. H&E Staining and IHC Staining

Adipocyte tissues were harvested and fixed in 10% formalin and then embedded in paraffin for sectioning. Hematoxylin and eosin (HE) staining was done according to the standard procedure. For IHC, sections were deparaffinized and incubated in a 0.01 M citrate buffer (PH = 6.0) at 95 °C for 30 min to retrieve the antigen, then incubated with 1% BSA to close the antigen. After that, sections were incubated with a primary antibody (Anti-UCP1 antibody, Abcam #ab10983, 1:100; Anti-PRDM16 antibody, Abcam# ab106410, 1:100 or Anti-CD68 antibody Abcam# ab125212, 1:100) for 1 h. After three times of washing, sections were incubated with an HRP-labeled secondary antibody IgG (1:200) for 1 h at room temperature. DAB agent was added, followed by hematoxylin staining.

### 4.11. OGTT and Insulin Test

Before the oral glucose tolerance test (OGTT), mice were fasted for 6 h with free access to water. Then, the mice were oral gavaged with 1 g/kg of glucose, and the levels of blood glucose were measured using an Accu-Check Performa (Roche Applied Science, Mannheim, Germany) at 0, 15, 30, 60, 90 and 120 min. Serum insulin levels were measured using an ultra-sensitive mouse insulin kit (Crystal Chem. Inc., Elk Grove Village, IL, USA).

### 4.12. Total RNA Isolation and Quantitative Real-Time PCR (qPCR)

Total RNA was isolated from cells or tissues using an RNA isolation kit (R693402, Omega Bio-Tek, GA, USA). The first strand cDNA was reverse-transcribed by a TAKARA reverse transcription kit. qPCR was performed with SYBR premix (Roche) on a CFX96™ Real-Time PCR Detection System (Bio-Rad). The relative mRNA expression level was normalized by β-Actin. The sequences of the primers used are listed in Supplementary Table S1.

### 4.13. Statistical Analysis

The results in this study are reported as the mean ± standard error (SD). Unpaired two-tailed Student’s *t*-test was used for comparisons between two groups. Analysis of variance (ANOVA) with Tukey’s post-test was used to compare values among three or four experimental groups.

## Data Availability

All data are listed in tables or presented in figures in the main text or Supplementary Materials.

## Supplementary Materials

The following supporting information can be downloaded at: https://www.mdpi.com/article/10.3390/ijms23126563/s1.
