# Supplementary material for: DEPP Deficiency Contributes to Browning of White Adipose Tissue
Source: Int J Mol Sci. 2022 Jun 12;23(12):6563. doi: 10.3390/ijms23126563 (PMC9223522; doi:10.3390/ijms23126563)
Supplement: Supplementary file 1 [file ijms-23-06563-s001.zip › ijms-1751965-supplementary.pdf]

Supplementary Information

DEPP deficiency contributes to browning of white adipose tissue

Fusheng Guo[1*], Yanlin Zhu[1*], Yaping Han[1], Xuhui Feng[1], Zhifu Pan[1], Ying He[2], Yong Li[1,#], Lihua Jin[1,3,#]

[*] These authors contributed equally to this work.
[#] Correspondences: yongli@xmu.edu.cn (Y.L.); lihjin@coh.org (L.J.)

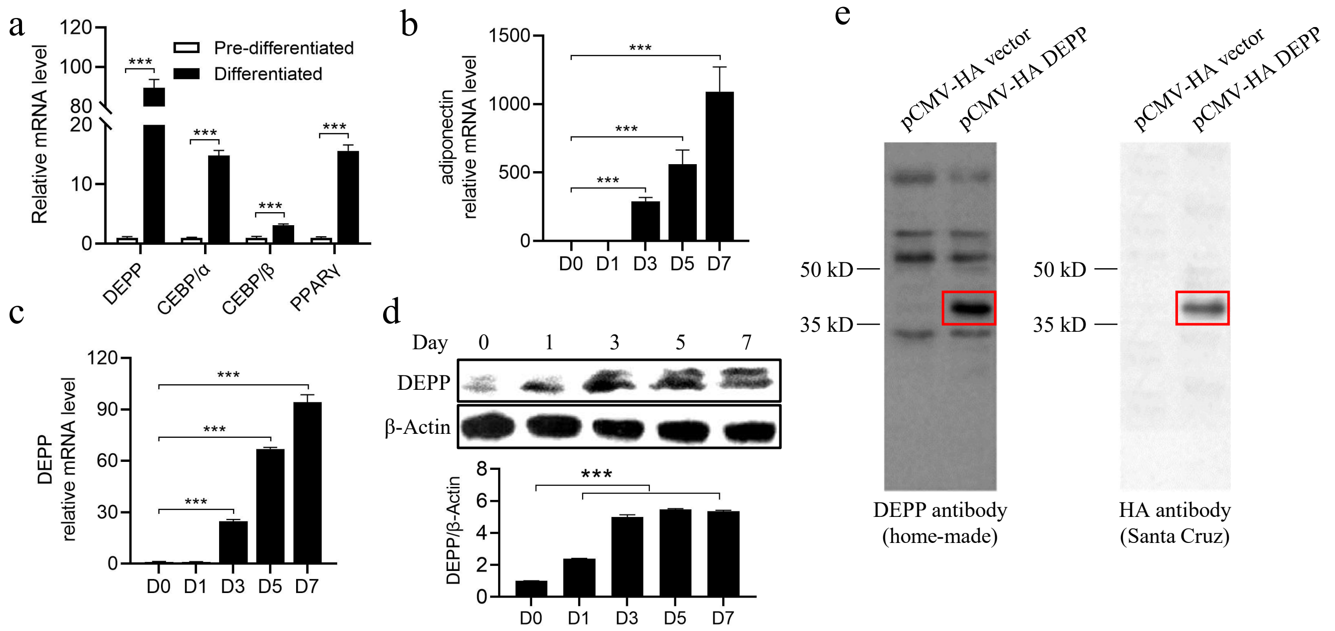

Supplemental Figure S1 DEPP expression increased in a differentiation-dependent manner in 3T3-LI cell. (**a**) Relative mRNA levels of DEPP and adipocyte differentiation - related genes. (**b-c**) Dynamic mRNA levels of Adiponectin (**b**) and DEPP (**c**) during adipocyte differentiation. (**d**) Western blot analysis and quantitative level of DEPP protein during differentiation in 3T3-L1 cell. (**e**) Evaluation of homemade anti-DEPP antibody. pCMV-HA-DEPP was transfected into HEK293T, and the cell lysate was used for Western blot analysis comparing homemade anti-DEPP antibody and commercial available anti-HA antibody, respectively. The band labeled in the red box were DEPP bands. In (**a**)**,** the results are presented as the means ± SD, Two-tailed Student's t-test, ***P < 0.001. In **b**, **c** and **d**, the results are presented as the means ± SD, One-way ANOVA with Tukey's post-hoc test, ***P < 0.001.

mouse-DEPP-siRNA-1	AAAA GGAAGAAACCACAGCACATCG TTGGATCCAA CGATGTGCTGTGGTTTCTTCC
mouse-DEPP-siRNA-2	AAAA GGGCTTCCTTTAGAGGAGACA TTGGATCCAA TGTCTCCTCTAAAGGAAGCCC
mouse-DEPP-siRNA-3	AAAA GCTTCCTTTAGAGGAGACAGT TTGGATCCAA ACTGTCTCCTCTAAAGGAAGC
mouse-DEPP-siRNA-4	AAAA GGAGACAGTCTTTACCCATCT TTGGATCCAA AGATGGGTAAAGACTGTCTCC
mouse-DEPP-siRNA-6	AAAA GTCTTTACCCATCTAGACTCC TTGGATCCAA GGAGTCTAGATGGGTAAAGAC
mouse-DEPP-siRNA-7	AAAA GCAGTATCCTAGGTACTCTCT TTGGATCCAA AGAGAGTACCTAGGATACTGC
mouse-DEPP-siRNA-9	AAAA GACAGTCTAGCTCCCACAATG TTGGATCCAA CATTGTGGGAGCTAGACTGTC
mouse-DEPP-siRNA-10	AAAA GCTCCCACAATGCGACTTTGA TTGGATCCAA TCAAAGTCGCATTGTGGGAGC

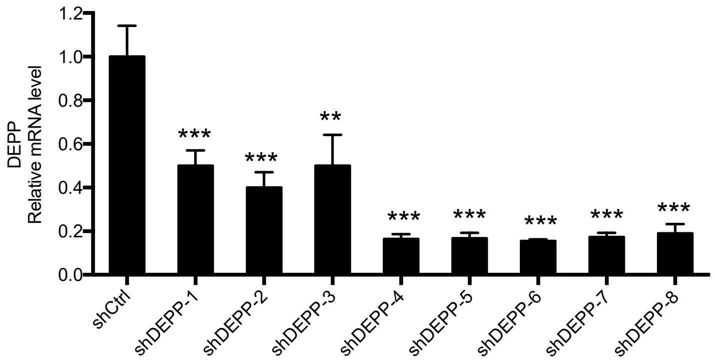

Supplemental Figure S2 shRNA-DEPP sequences and silence efficacy identification. The sequences designed for DEPP silence were listed. The silence efficacies of DEPP were determined by Q-PCR. n = 3 biological replicates, and the results are presented as the means ± SD, One-way ANOVA with Tukey's post-hoc test, **P < 0.01, ***P < 0.001 vs shCtrl.

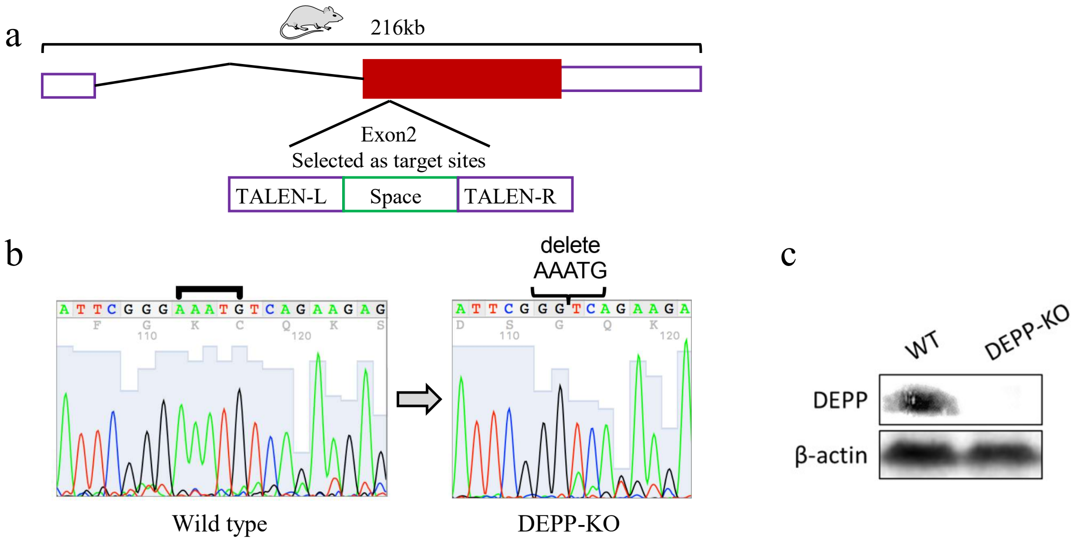

Supplemental Figure S3 DEPP knockout mice establishment in C57BL/6. A. TALENs technology was used to create DEPP-knockout mice through inducing the deletion of five bases on the second exon of Depp gene. B. Genomic DNA was isolated from mouse tails. Target site was amplified by PCR and then sequenced. The left image indicates the sequencing from wild type mice and the right image indicates the deletion of AAATG motif in *Depp* KO mice. This deletion results in a frameshift to achieve DEPP deficiency *in vivo*. C. Western blot analysis on the embryonic fibroblasts isolated from *Depp*$^{-/-}$ mice.

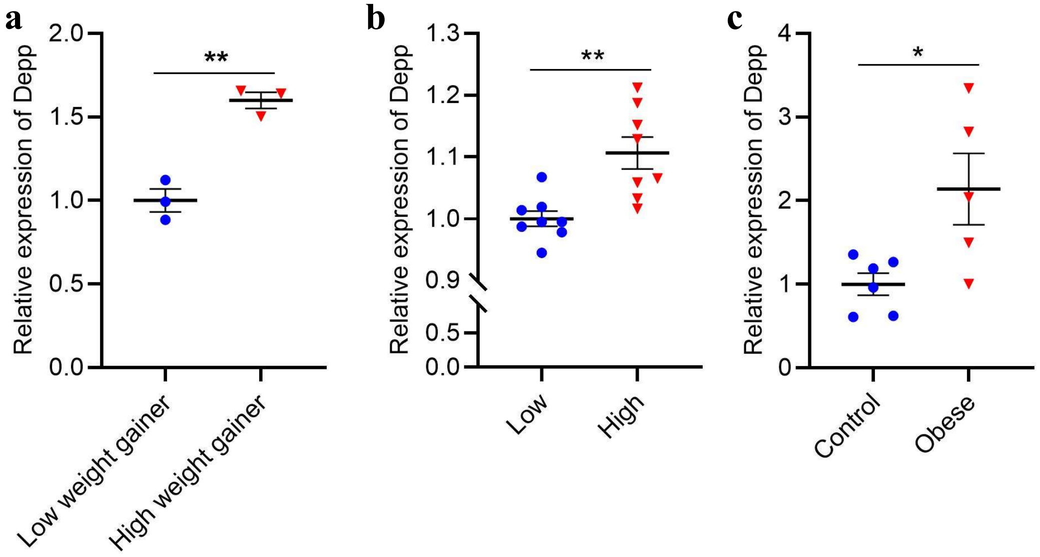

Supplementary Figure S4 GEO profiles of DEPP in adipose tissue. Higher Depp expression level in (**a**) iWAT of mice under 4 week's high saturated fat diet feeding (GEO profile ID: 30164547), (**b**) iWAT of mice T2D model (GEO profile ID: 75593942), and (**c**) visceral adipose tissue of obese individuals (GEO profile ID: 30164547). Two-tailed Student's t-test: *P < 0.05, **P < 0.01.

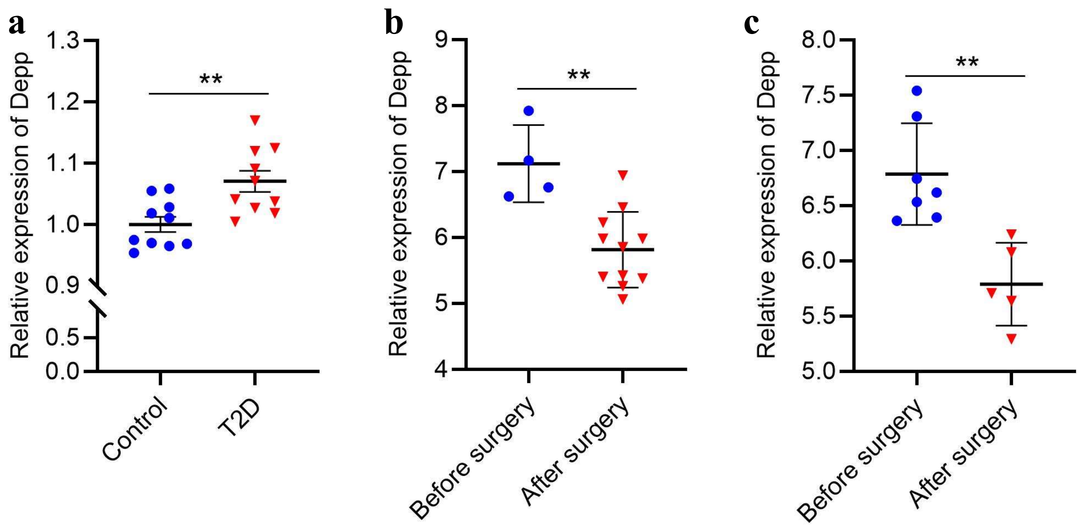

Supplementary Figure S5 GEO profiles of DEPP in muscle and liver tissue. (**a**) Higher Depp expression levels in skeletal muscle myotube in T2D individuals (GEO profile ID: 64654601). (**b-c**) Downregulated Depp expression in liver of individuals with obese (b) or obese with NAFLD (c) after bariatric surgery (GEO profile ID: 7933203) .

Supplementary Table S1. The sequences of primers used in Q-PCR.

Gene name	Forward primer (5'-3')	Reverse primer (5'-3')
GAPDH	GCCTTCCGTGTTCCTACCC	TGCCTGCTTCACCACCTTC
DEPP	TGAGCACTCTCTGGGAAGAAAAC	GATCACTGGGAGGTGCAAATAGA
CEBPa	CCAAGAAGTCGGTGGACAAGA	CGGTCATTGTCACTGGTCAACT
CEBPβ	CGCCTTATAAACCTCCCGCT	TGGCCACTTCCATGGGTCTA
Adiponectin	GAATCATTATGACGGCAGCAC	CCAGATGGAGGAGCACAGAG
Cidea	ATCACAACTGGCCTGGTTACG	TACTACCCGGTGTCCATTTCT
Cox8b	GAACCATGAAGCCAACGACT	GCGAAGTTCACAGTGGTTCC
PGC-1α	AGCCGAGGACACGAGGAAAG	TGGCCTGAATCTGTGGAAGAAC
PPARγ	CATTCTGGCCCACCAACTTC	TCAAAGGAATGCGAGTGGTCTT
PRDM16	CAGCACGGTGAAGCCATTC	GCGTGCATCCGCTTGTG
Resistin	AAGAACCTTTCATTTCCCCTCCT	GTCCAGCAATTTAAGCCAATGTT
UCP1	CACCTTCCCGCTGGACACT	CCCTAGGACACCTTTATACCTAATGG
WDNM1	TCAACCCAGTCAGAGCCAAC	GCCCAGGCAGTAGTCATTGT
Agt	GCACCCTGGTCTCTTTCTACC	TGTGTCCATCTAGTCGGGAGG
Itga6	GGCGACCGAGGCCAAG	GTGTCCAGGTTGAAGGCTGT
PPARα	GCGTACGGCAATGGCTTTAT	GAACGGCTTCCTCAGGTTCTT

Supplementary Table S2. Raw data for Supplementary Fig. 1b-c.

Actin	D0	16.07	16.20			
		16.41				
		16.12				
	D1	16.09				
		16.16				
		15.85				
	D3	16.35				
		16.24				
		15.73				
	D5	16.24				
		16.15				
		15.76				
	D7	16.24				
		16.26				
		16.32				
DEPP	D0	32.45	32.64	-0.06	1.044877153	1.052975
		32.31		-0.54	1.457335791	
		33.17		0.61	0.656712278	
	D1	32.58		0.05	0.968170696	1.124152
		32.2		-0.40	1.322560146	
		32.18		-0.11	1.081724666	
	D3	28.26		-4.53	23.1563078	24.80093
		27.95		-4.73	26.59961268	
		27.55		-4.62	24.64688356	
	D5	26.65		-6.03	65.49592909	66.88611
		26.49		-6.10	68.75216947	
		26.15		-6.05	66.41021819	
	D7	26.04		-6.64	99.96376492	93.91833
		26.29		-6.41	85.23259388	
		26.17		-6.59	96.55863211	
Adiponectin	D0	25.94	26.01	0.06	0.959264119	1.000577
		26.22		0.00	1	
		25.87		-0.06	1.042465761	
	D1	27.74		1.84	0.279321785	0.416073
		26.86		0.89	0.539614118	
		26.88		1.22	0.429282718	
	D3	18.19		-7.97	250.7315962	290.4817
		17.93		-8.12	278.2041248	
		17.12		-8.42	342.509454	
	D5	16.48		-9.57	760.0760682	561.0726
		17.24		-8.72	421.6786008	
		16.6		-8.97	501.4631924	
	D7	15.59		-10.46	1408.554822	1086.504
		16		-10.07	1074.909884	
		16.53		-9.60	776.0468821	
